# HiCMamba: Enhancing Hi-C resolution and identifying 3D genome structures with state space modeling

**DOI:** 10.1371/journal.pcbi.1014057

**Published:** 2026-03-24

**Authors:** Minghao Yang, Zhi-An Huang, Zhihang Zheng, Yuqiao Liu, Shichen Zhang, Pengfei Zhang, Hui Xiong, Shaojun Tang

**Affiliations:** 1 Artificial Intelligence Thrust, Hong Kong University of Science and Technology (Guangzhou), Guangzhou, China; 2 Department of Computer Science, City University of Hong Kong (Dongguan), Dongguan, China; 3 Bioscience and Biomedical Engineering Thrust, Hong Kong University of Science and Technology (Guangzhou), Guangzhou, China; 4 Department of Computer Science and Engineering, The Hong Kong University of Science and Technology, Hong Kong SAR, China; 5 Department of Biostatistics, Virginia Commonwealth University, Richmond, Virginia, United States of America; 6 Massey Comprehensive Cancer Center, Bioinformatics Shared Resource Core, Virginia Commonwealth University, Richmond, Virginia, United States of America; Korea Institute for Advanced Study, KOREA, REPUBLIC OF

## Abstract

Hi-C technology measures genome-wide interaction frequencies, providing a powerful tool for studying the 3D genomic structure within the nucleus. However, high sequencing costs and technical challenges often result in Hi-C data with limited coverage, leading to imprecise estimates of chromatin interaction frequencies. To address this issue, we present a novel deep learning-based method HiCMamba to enhance the resolution of Hi-C contact maps using a state space model. We adopt the UNet-based auto-encoder architecture to stack the proposed holistic scan block, enabling the perception of both global and local receptive fields at multiple scales. Experimental results demonstrate that HiCMamba outperforms state-of-the-art methods while significantly reducing computational resources. Furthermore, the 3D genome structures, including topologically associating domains (TADs) and loops, identified in the contact maps recovered by HiCMamba are validated through associated epigenomic features. Our work demonstrates the potential of a state space model as foundational frameworks in the field of Hi-C resolution enhancement. The data and source code used in this work are available at GitHub: https://github.com/myang998/HiCMamba.

## Author summary

Understanding the 3D structure of chromosomes within the cell nucleus is fundamental to deciphering gene regulation. A key technology known as Hi-C allows us to map this 3D genome, but obtaining high-resolution data is often hindered by high costs and technical challenges. This results in the prevalence of low-resolution data, which obscures the fine-scale structural details essential for analysis. To address this limitation, we have developed HiCMamba, a novel deep-learning framework that, for the first time, leverages a state space model for this task. In this study, we introduce HiCMamba to computationally enhance low-resolution Hi-C contact maps to high-resolution quality. Our method is designed to effectively capture both the long-range and local chromatin interactions that define 3D genome architecture. Experimental results demonstrate that HiCMamba outperforms existing state-of-the-art methods, achieving superior accuracy while significantly reducing computational resource requirements. We show that contact maps enhanced by HiCMamba lead to more precise identification of critical genomic structures, including TADs and chromatin loops. Our work provides researchers with a more powerful and accessible tool for high-resolution 3D genome analysis. These advancements not only facilitate a deeper understanding of the interplay between genome structure and cell-specific gene regulation but also establish the potential of state space models as a foundational framework for future innovations in genomics research.

## Introduction

Nuclear genomes house the majority of genetic information essential for determining the phenotype of cells, tissues, and organisms [1]. Within the nucleus, chromosomes are intricately folded and organized in three-dimensional space, allowing various chromosomal loci to interact with one another [2,3]. This 3D genome architecture plays a critical role in regulating gene expression and maintaining cellular homeostasis [4–6]. High-throughput chromosome conformation capture (Hi-C) [7] has become a powerful method for measuring the 3D genome structure, enabling the discovery of inherent hierarchical topological features such as A/B compartments [7], topologically associating domains (TADs) [8], and loops [9]. Low-resolution Hi-C data result in blurred TADs and loops, highlighting the necessity of using high-resolution Hi-C sequencing data to accurately identify these spatial patterns. In recent years, high-resolution Hi-C data (e.g., 10kb and 40kb) [8,9] have become available, enabling more efficient and accurate identification of TADs and loops. High-resolution Hi-C data are increasingly in demand among researchers investigating the intricate 3D structures of chromosomes. Nevertheless, owing to technical constraints and high sequencing costs, most publicly available high-resolution Hi-C data are derived from labor-intensive and time-consuming experiments. Consequently, there is an urgent need to develop computational methods to improve the resolution of Hi-C data. Advancement of deep-learning methods for in-silico image refinement made improvement of low-resolution HiC data possible. Recently, computational tools have been proposed to expedite the enhancement of HiC data resolution.

The Hi-C data are generally displayed as an n×n contact matrix, with the chromosome segmented into *n* equally sized bins. Specifically, the value of each cell in the matrix reflects the frequency of interaction between two genomic loci. The high resolution of Hi-C contact matrices, often exceeding 10,000 bins for a single human chromosome (e.g., a 100 Mb chromosome with 10kb bin width), poses a significant challenge for current deep learning methods. These methods typically partition the matrices into non-overlapping blocks and downsample the original high-coverage maps, resulting in lower-resolution. Low-coverage Hi-C contact maps, generated through downsampling, are then fed into the deep learning models to reconstruct the original high-coverage maps. Existing computational methods for enhancing low-coverage Hi-C contact matrices can be generally categorized into two groups: the traditional convolutional neural network (CNN)-based and the generative adversarial network (GAN)-based methods. Traditional CNN-based methods, such as HiCPlus [10] and HiCNN [11], employ multiple CNN blocks to predict high-coverage contact maps from low-coverage inputs. However, their reliance on mean square error (MSE) loss often leads to over-smoothed and blurry predictions [12]. Moreover, several GAN-based models, e.g., HiCSR [13] and HiCARN [14], have been proposed to generate high-coverage contact maps. These methods include a generator that transforms low-coverage contact maps into high-coverage contact maps, and a discriminator that takes both the generated and real contact maps as input, classifying them as either real or fake.

While previous methods for enhancing Hi-C contact maps have shown promise, they suffer from several limitations for their heavy reliance on CNNs. CNNs, with their inherent local receptive fields, struggle to capture the crucial long-range dependencies present in Hi-C data. In addition, these methods, coupled with the need for deep architectures to compensate, often results in high computational complexity and cost. Besides, GAN-based methods often face convergence issues for the adversarial training between the generator and discriminator.

To tackle these challenges, we propose a Mamba-based auto-encoder framework HiCMamba, leveraging state space model [15] to infer the high-coverage Hi-C contact maps based on UNet architecture [16]. HiCMamba incorporates a novel holistic scan block within each layer to effectively capture multi-scale features. This block consists of a two-dimensional selective scan (SS2D) module and a locally-enhanced feedforward neural network (LEFN). The SS2D module achieves a global receptive field with linear complexity by using a four-way sequential scanning strategy based on a state space model (SSM). The LEFN consisting of multi-layer CNN captures local information of neighborhood pixels.

Extensive experiments on Hi-C datasets demonstrate that HiCMamba outperforms state-of-the-art methods in both effectiveness and generalization while significantly reducing computational cost. Notably, HiCMamba achieves these high-quality recovery results with only 25% of the computational cost compared to the runner-up method. HiCMamba exhibits global receptive fields, in contrast to other methods that are restricted to local receptive fields. Furthermore, HiCMamba-recovered contact maps showcase accurate 3D genome structure identification, such as TADs and loops. Finally, we find that the 3D genome structure, along with epigenomic features such as ChIP-seq, methylation, and super-enhancer (SE) regions, plays an integral role in regulating gene expression.

## Materials and methods

### Hi-C Datasets and data preprocessing

The raw Hi-C data consists of an n×n interaction frequency contact matrix, representing the all-versus-all interaction mapping of fragments within a chromosome [7]. Each matrix entry indicates the interaction frequency between a pair of fragments, where *n* represents the number of fragments in a chromosome at a given Hi-C data resolution.

In this study, the high-coverage Hi-C data of 10kb resolution are downloaded from GEO under accession number GSE63525 [17]. Two widely investigated cell lines (i.e., GM12878 and K562) are employed to assess the effectiveness of HiCMamba. Following previous works [14,18], we preprocess the 10-kb resolution Hi-C data through normalization, down-sampling, and data division. Specifically, KR normalization [19] is applied to the high-resolution Hi-C contact maps, derived from paired-end sequencing reads with a mapping quality greater than 30. Low-coverage contact maps are then generated by downsampling the normalized data at a ratio of 1/16, simulating the lower resolutions achieved at reduced sequencing depths in practice [10]. The contact maps are subsequently split into non-overlapping 40×40 sub-matrices. Low-coverage and high-coverage (target) contact maps are created by concatenating these sub-matrices, with the high-coverage maps skipping the downsampling step.

### The HiCMamba algorithm

**Fig 1:**
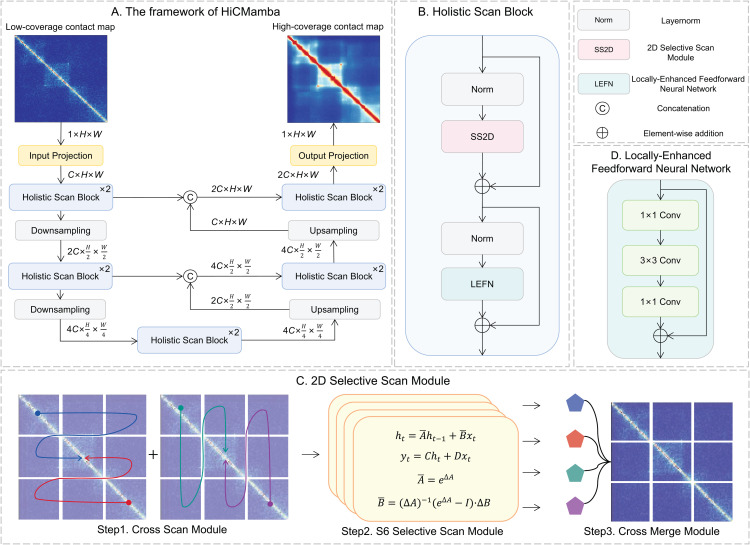
Overview of the HiCMamba algorithm. **(A)** The framework of HiCMamba. The workflow begins with an input projection layer that extracts shallow features from the low-coverage Hi-C contact map (input). These extracted features are then input into a UNet-based auto-encoder architecture, which utilizes our proposed holistic scan block. This block facilitates the feature extraction and reconstruction at multiple scales. In the final stage, these refined features pass through an output projection layer to reconstruct the final high-coverage Hi-C contact map. **(B)** The architecture of the proposed holistic scan block, structured as Norm → SS2D → Norm → LEFN, follows the design of the Transformer block. **(C)** Diagram of the SS2D module. First, the input features are flattened along four distinct scanning paths. Each path is processed independently by an individual S6 selective scan module. Finally, the outputs from each path are combined to reconstruct the 2D feature map. **(D)** Structure illustration of LEFN.

In this work, our aim is to enhance the resolution of Hi-C data given a low-resolution contact map. Here, we develop a multi-scale framework HiCMamba, leveraging the state space model [15] within a hierarchical UNet framework to capture long-range dependencies while reducing computational costs. As shown in Fig 1A, HiCMamba utilizes a UNet-based auto-encoder architecture that incorporates holistic scan blocks to extract features across multiple scales. Specifically, the proposed HiCMamba comprises an input projection layer, an encoder, a bottleneck layer, a decoder, and an output projection layer. Convolutional layers in the input and output projection layers extract the low-level features and reconstruct high-coverage contact maps, respectively. Two holistic scan blocks, for the extraction of global and local representations using SS2D and LEFN, are used in the bottleneck layer and in each layer of the encoder and decoder. Given a single channel low-coverage Hi-C contact map $X_{in}\in {\mathbb{R}}^{1\times H\times W}$, a convolutional layer is first applied in the input projection layer to extract the shallow representations $E_{1}\in {\mathbb{R}}^{C\times H\times W}$. *C* represents the predefined feature dimension, while *H* and *W* denote the height and width of the input contact map, respectively. Next, *E*_1_ is iteratively passed through two layers of holistic scan blocks along with downsampling, resulting in $E_{2}\in {\mathbb{R}}^{2C\times \frac{H}{2}\times \frac{W}{2}}$ and $E_{3}\in {\mathbb{R}}^{4C\times \frac{H}{4}\times \frac{W}{4}}$. A bottleneck stage composed of holistic scan blocks is incorporated at the end of the encoder. For feature reconstruction, the proposed decoder also comprises two stages, with upsampling following holistic scan blocks. The features *E*_*i*_ from the *i*-th encoder stage are concatenated with *D*_*i*_ from the previous decoder stage using skip connections. Where *D*_*i*_ is the output of the *i*-th decoder stage with the same shape as *E*_*i*_. Finally, the output of the decoder is fed into the output projection layer to obtain the high-cover contact map. The *L*_1_ loss is adopted for training, which has been demonstrated to be less susceptible to over-smoothing [20]. The definition of *L*_1_ loss is provided as follows:


$$L_{1}(\hat{y},y)=\frac{1}{HW}\sum_{i=0}^{H-1}\sum_{j=0}^{W-1}|\hat{y}(i,j)-y(i,j)|$$ (1)

Fig 1B showcases the backbone of the holistic scan block, structured as Norm → SS2D → Norm → LEFN, similar to the design of the Transformer block [21]. Here, Norm  denotes layer normalization [22]. The SS2D module (Fig 1C) enables a comprehensive scan of information from different directions with low time complexity, and the LEFN module (Fig 1D) leverages multiple CNNs to facilitate the inception of a local receptive field. The combination of SS2D and LEFN effectively captures both global and local receptive fields simultaneously. The details of the Mamba-based holistic scan block, the SS2D module, and the LEFN module are described in the following section.

#### Mamba-based holistic scan block.

We propose a novel holistic scan block (Fig 1B) to address the limitations of traditional CNN and GAN-based methods, which struggle to capture long-range dependencies, entail high computational costs, and often face training instability.

First, the SS2D module within the block effectively captures long-range dependencies in Hi-C data. This is achieved by a multi-path scanning strategy coupled with robust sequential modeling capabilities of the S6 block. By gathering information from multiple directions and leveraging the S6 block’s advanced sequence modeling, the holistic scan block accurately represents distant genomic loci interactions, ensuring comprehensive global information extraction.

Second, unlike computationally expensive deep CNN structures, our approach leverages the linear time complexity of the state space model for efficiency. The Mamba-based holistic scan block performs efficient state transitions, requiring significantly less computational power compared to deep convolutional layers. This inherent efficiency eliminates the need for complex, computationally demanding deep architectures.

Third, by avoiding adversarial training, HiCMamba overcomes the convergence challenges often encountered in GAN-based methods. The robust modeling capabilities of SS2D and LEFN within the holistic scan block ensure stable and consistent performance throughout the training process.

Moreover, state space model does not explicitly compute dependencies among pixels and is thus not proficient in capturing local information [23]. To recognize the importance of local context for accurate Hi-C resolution enhancement, our holistic scan block incorporates the LEFN module. This module complements the global dependency capture of the SS2D module, enabling detailed reconstruction of high-resolution contact maps by leveraging information from neighboring pixels.

We structure the holistic scan block following the design of the Transformer [21] block, improving gradient flow and training stability by applying layer normalization before the SS2D and LEFN. Overall, the proposed holistic scan block effectively captures both long-range dependencies and valuable local context by combining the strengths of state space modeling and convolutional operations. The SS2D and LEFN components are detailed in the following subsections.

#### 2D selective scan module.

We introduce the 2D Selective Scan (SS2D) module to effectively capture global receptive fields [24], ensuring comprehensive and efficient feature extraction from the genomic interaction data. As illustrated in Fig 1C, SS2D comprises three steps: cross scan module, S6 selective scan module [15], and cross merge module.

First, the cross scan module initially transforms the input contact map into sequences along four unique traversal paths (i.e., from top-left to bottom-right, bottom-right to top-left, top-right to bottom-left, and bottom-left to top-right). This method is particularly suited to the complex nature of Hi-C data, ensuring a thorough scan of interaction frequencies. The generated sequences are then fed into the S6 selective scan module for a detailed representation of the contact map.

Second, the S6 selective scan module, a variant of the state space model with a selective scan mechanism, functions as a linear time-invariant system. Mathematically, it maps the input state *x*(*t*) to the output state *y*(*t*) via the hidden state *h*(*t*), which is typically represented by linear ordinary differential equations as follows:


$$h^{\prime }(t)=Ah(t)+Bx(t)$$ (2)

y(t)=Ch(t) (3)

where *A*, *B*, and *C* are learnable parameter matrices. *h*’(*t*) represents the derivative of the hidden state *h*(*t*) at time step *t*. *A* retains historical information, shaping the influence of the prior hidden state on the current hidden state, while *B* quantifies the impact of the input *x*(*t*) on the hidden state. *C* delineates the transformation of the hidden state into the output.

To be incorporated into deep learning models, continuous-time state space models must be discretized beforehand, which can be obtained using the zeroth-order hold method as follows:


$$h_{t}=\overset{―}{A}h_{t-1}+\overset{―}{B}x_{t}$$ (4)

$$y_{t}=Ch_{t}$$ (5)

$$\overset{―}{A}=e^{\Delta A}$$ (6)

$$\overset{―}{B}=(\Delta A{)}^{-1}(e^{\Delta A}-I)\cdot \Delta B$$ (7)

where *h*_*t*_ is the discrete hidden state at time step *t*, while $h_{t-1}$ represents the hidden state of the previous step. The discrete input and output representation at time step *t* are denoted as *x*_*t*_ and *y*_*t*_, respec*t*ively. The continuous parameters *A* and *B* are converted to discrete parameters $\overset{―}{A}$ and $\overset{―}{B}$ using the zeroth-order hold method with a timescale parameter Δ.

The iterative calculation of *y* can be accelerated using parallel global convolutional operation as follows:


$$y=x⊙\overset{―}{K}$$ (8)

$$\overset{―}{K}=(C\overset{―}{B},C\overset{―}{A}\overset{―}{B},\ldots ,C{\overset{―}{A}}^{T-1}\overset{―}{B})$$ (9)

where ⊙, $\overset{―}{K}$, and *T* represent the global convolutional operation, the convolutional kernel, and the total number of pixels, respectively. The pseudo code for S6 selective scan module is provided in Algorithm 1.

Finally, the cross merge module combines and merges the sequence representations extracted by the four S6 modules to reconstruct the high-coverage contact map, maintaining the same size as the input low-coverage contact map.

The adaptation of SS2D to Hi-C data is crucial for addressing the challenges posed by genomic interactions, which enables HiCMamba to accurately capture long-range dependencies between pixels and complex chromatin interactions with high efficiency. This advancement improves the resolution of Hi-C contact maps with reduced computational costs, providing a powerful tool for 3D genomic data analysis.

**Algorithm 1 Pseudo code for S6 selective scan module**

**Input:** *x*, the raw feature maps

**Output:** *y*, the refined feature maps

 1: **Params:** *W*_*A*_, *W*_*B*_, *W*_*C*_, the learnable parameters for linear transformations

 2: **Params:** Δ, the timescale parameter for discretization

 3: **Step 1: Linear Transformations**

 4: $A,B,C\leftarrow \text{Linear}(x,W_{A}),\text{Linear}(x,W_{B}),\text{Linear}(x,W_{C})$

 5: **Step 2: Exponential Calculations and Discretization**

 6: $\overset{―}{A}\leftarrow \exp(\Delta \cdot A)$

 7: $\overset{―}{B}\leftarrow (\Delta \cdot A{)}^{-1}\cdot (\exp(\Delta \cdot A)-I)\cdot B$

 8: **Step 3: Global Convolutional Operation for Acceleration**

 9: $\overset{―}{K}\leftarrow (C\overset{―}{B},C\overset{―}{A}\overset{―}{B},\ldots ,C{\overset{―}{A}}^{T-1}\overset{―}{B})$

 10: $y\leftarrow x⊙\overset{―}{K}$

 11: **return** *y*

#### Locally-Enhanced Feedforward Neural Network Moudle.

Previous research [25,26] has highlighted the limited ability of feedforward neural networks to utilize local context effectively. Given the importance of neighboring pixels in Hi-C contact map recovery, LEFN is designed to enable a local receptive field. As shown in Fig 1D, we first apply a 1×1 convolution layer to each token to enhance the feature dimension. A 3×3 convolution layer is then used to capture local information. Finally, the features are processed through another 1×1 convolution layer to reduce the channels for matching match the input dimension. Each CNN layer is followed by a GELU activation layer [27].

### Structure Weighted Scoring

We propose a cell-type specific structure weighted score to systematically measure the correlation between enhanced genomic structures (i.e., loops and TAD boundaries) and cell-type-specific regulatory features (e.g., super-enhancers or CTCF binding sites). First, the proportion of structures associated with specific regulatory features for each cell line is calculated as follows:


$$P_{l}^{s}=\frac{A_{l}^{s}}{N_{l}}$$ (10)

where l∈{GM12878,K562} and s∈{GM12878,K562} denote the cell lines for the identified structures and the regulatory features, respectively. $A_{l}^{s}$ represents the number of *l*-specific structures associated with *s*-specific regulatory features, and *N*_*l*_ is the total number of structures identified in the cell *l*ine *l*. Then, the structure weighted score $W_{l}^{s}$ is calculated as follows:


$$W_{l}^{s}=\frac{P_{l}^{s}}{P_{GM12878}^{s}+P_{K562}^{s}}$$ (11)

This score represents the relative contribution of each cell line’s regulator-associated structures to the total across both cell lines, providing a quantitative measure of cell-type specificity.

### Experimental setup

HiCMamba is developed using Python and PyTorch, and executed on the Ubuntu platform with a Tesla V100 GPU. We follow previous studies for data partitioning [14]: chromosomes 2, 6, 10, and 12 are used for validation; chromosomes 4, 14, 16, and 20 for testing; and the remaining chromosomes for training. HiCMamba handles the low-resolution input at a 40×40 resolution. The number of holistic scan blocks in the bottleneck layer, as well as in each layer of the encoder and decoder, is set to two. The predefined feature dimension *C* is set to 32. The number of neurons of input features of bottleneck layer, the *i*-th layer of encoder, and the *i*-th layer of decoder are 4*C*, i×C, and i×2C, respectively. The LEFN is composed of three layers of CNNs with kernel sizes of 1×1, 3×3, and 1×1. We set the batch size to 64 and train HiCMamba using the Adam optimizer [28], with a learning rate of 1e-4 and momentum parameters $\beta_{1}$ and $\beta_{2}$ set to 0.9 and 0.999, respectively.

## Results

### HiCMamba demonstrates efficacy in recovering high-coverage Hi-C contact maps

HiCMamba is benchmarked with three state-of-the-art methods, involving HiCNN [11], HiCSR [13], and HiCARN [14]. Evaluations are conducted on the preprocessed datasets from GM12878 and K562 cell lines. To ensure a fair comparison, all methods are implemented using their default parameters as reported in their respective publications. We evaluate model performance using a combination of local metrics, including Pearson correlation coefficient (PCC) and Spearman rank correlation coefficient (SRCC), as well as global structural metrics, namely HiC-Spector [29], GenomeDISCO [30], and Compartment score [7]. Table 1 illustrates the test performance of the compared methods on GM12878 and K562 datasets, respectively. HiCMamba achieves the highest performance in 8 out of the 10 comparisons across the two datasets. For instance, on the K562 dataset, our method surpasses all state-of-the-art baselines across every metric evaluated, exceeding the second-best method by 3.5% in PCC, 7.1% in SRCC, and 5.4% in Compartment score. These comprehensive evaluations confirm that the proposed holistic scan block effectively recovers both fine-grained interactions and the essential global topological organization of the 3D genome.

**Table 1:** Quantitative comparison of different methods on GM12878 and K562 datasets. Results highlighted in bold and underlined represent the best and second-best, respectively.

Dataset	Methods	PCC	SRCC	GenomeDISCO	HiC-Spector	Compartment
GM12878	HiCSR [13]	0.5573	0.5050	0.9502	0.9468	0.5053
	HiCNN [11]	0.5752	0.5322	0.9552	0.9488	**0.5168**
	HiCARN [14]	0.5867	0.5242	0.9584	**0.9611**	0.5061
	HiCMamba (Ours)	${\text{0.6113}}_{+4.2\%}$	${\text{0.5597}}_{+5.2\%}$	${\text{0.9619}}_{+0.4\%}$	${\underset{―}{0.9531}}_{-0.8\%}$	${\underset{―}{0.5107}}_{-1.2\%}$
K562	HiCSR [13]	0.4288	0.3416	0.8828	0.8350	0.5485
	HiCNN [11]	0.4964	0.3919	0.9172	0.9023	0.5203
	HiCARN [14]	0.5042	0.3955	0.9260	0.9131	0.6462
	HiCMamba (Ours)	${\text{0.5219}}_{+3.5\%}$	${\text{0.4236}}_{+7.1\%}$	${\text{0.9310}}_{+0.5\%}$	${\text{0.9231}}_{+1.1\%}$	${\text{0.6810}}_{+5.4\%}$

**Fig 2:**
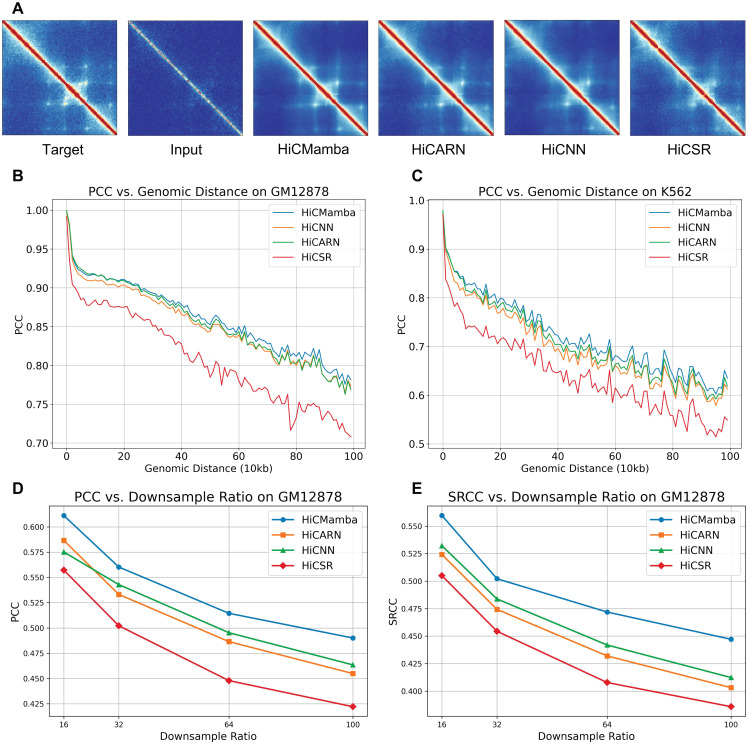
HiCMamba enhances the contact matrix. **(A)** Contact maps for a 1-Mb genomic region of Chromosome 14 (32Mb-33Mb) from the GM12878 dataset. The first column represents the full-coverage map, the second column represents the low-coverage input, the third column shows the enhanced map generated by HiCMamba, and the remaining columns display maps from compared methods. **(B-C)** PCC between enhanced and ground-truth maps across varying genomic distances for GM12878 (Panel B) and K562 (Panel **C)**. **(D-E)** Performance comparison of different methods across varying downsampling ratios (1/16, 1/32, 1/64, and 1/100) on the GM12878 dataset, evaluated by PCC (Panel D) and SRCC (Panel **E)**.

Fig 2A visually compares the full-coverage (target), low-coverage (input), and enhanced contact maps predicted by each compared method for a 1-Mb genomic region (chr14:32Mb–33Mb) on the GM12878 dataset. All enhanced contact maps show improvement over the low-coverage input, with HiCMamba and HiCARN exhibiting a greater ability to capture fine-scale structures such as loops. Fig 2B and 2C further highlight HiCMamba’s superior performance by illustrating the PCCs between predicted and ground-truth high-coverage contact maps for both datasets across various distance ranges. HiCMamba consistently outperforms existing methods, particularly in sparse regions of the contact map. Overall, the experimental results demonstrate the effectiveness of HiCMamba. The combination of the UNet architecture, state space models, and locally enhanced feedforward networks allows for efficient capture of both global and local features at multiple scales, leading to superior performance in Hi-C contact map enhancement.

We further evaluate the performance of HiCMamba under varying downsampling ratios (1/32, 1/64, and 1/100) on the GM12878 dataset investigate the impact of various resolutions on model performance. As shown in Fig 2D and 2E, both PCC and SRCC exhibit a monotonic decline as the input coverage decreases across all methods. Nevertheless, HiCMamba consistently outperforms state-of-the-art methods across all sparsity levels. Notably, at the extreme downsampling ratio of 100, HiCMamba maintains a PCC of 0.4901, whereas the runner-up, HiCARN, drops to 0.4635. We also evaluate how resolution scaling affects the PCCs between predicted and ground-truth contact maps across various distance ranges. As illustrated in S1A Fig, HiCMamba performs robustly across varying downsampling ratios. The results show that a minor decline in the short-to-medium range (< 200kb) for the 1/100 ratio compared to 1/16, which is consistent with the challenges of extreme data sparsity. This demonstrates that the global receptive field of the state space model offers superior resilience to data sparsity compared to local convolution-based approaches.

To investigate the limits of our method under pseudo-single-cell sparsity, we consider the 1/100 downsampling scenario—which effectively removes 99% of sequencing reads—as a proxy for pseudo-single-cell data. While HiCMamba consistently outperforms baseline methods on the GM12878 dataset despite the expected performance decline, we extend this stress test to the inherently sparser K562 cell line [9], where our experiments reveal a clear performance boundary. While all methods remain functional at a 1/64 downsampling ratio, the more extreme 1/100 ratio push several regions beyond their reconstruction limits. On chromosomes 4, 14, and 16, HiCMamba demonstrates greater resilience, achieving an average PCC of 0.39 compared to 0.37 for HiCARN. However, all methods fail to recover meaningful structures on chromosome 20. This is attributed to the fact that chromosome 20 is significantly sparser than others in the already low-coverage K562 cell line, causing the signal-to-noise ratio at 1/100 downsampling to drop below the critical threshold required for valid reconstruction and thus marking a practical lower bound for current computational approaches. Consequently, HiCMamba effectively leverages its combined global and local receptive fields to deliver optimal performance up to the physical limits of data recoverability.

### HiCMamba offers a global receptive field with lower time cost

**Fig 3:**
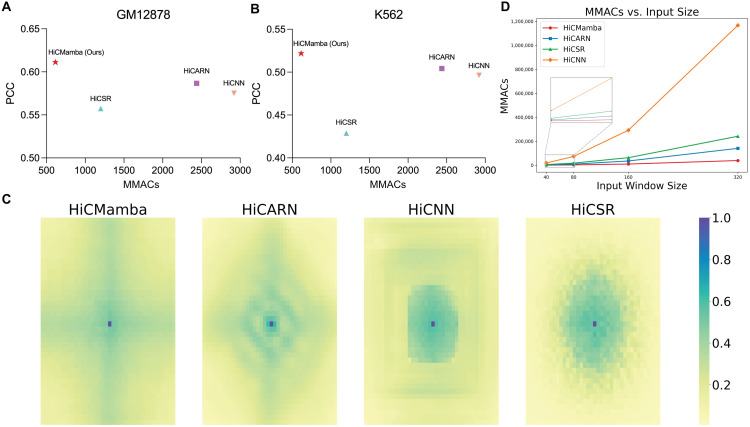
Performance, computational cost, and receptive field comparison of HiCMamba with state-of-the-art methods. **(A-B)** PCC versus MACs of HiCMamba and alternative tools on the GM12878 (A) and K562 (B) datasets, respectively. **(C)** Visualization of the effective receptive field (ERF) for HiCMamba compared to other state-of-the-art methods. **(D)** Comparison of computational complexity (MACs) of different methods across varying input window sizes.

In this section, we evaluate the computational efficiency of HiCMamba and other state-of-the-art methods using multiply-accumulate operations (MACs) as a measure of resource utilization. As depicted in Fig 3A and 3B, HiCMamba achieves the highest PCC while using only 25%, 21%, and 61% of the MACs compared to HiCARN, HiCNN, and HiCSR, respectively. These highlight HiCMamba’s ability to surpass the accuracy of existing methods with greater efficiency. Beyond computational efficiency, we analyze the effective receptive field [31] of each method, which represents the region within the input space that influences the activation of a specific output unit. A wider effective receptive field indicates a stronger capability to capture long-range dependencies and global context. Focusing on the central pixel, Fig 3C illustrates a key distinction: HiCMamba exhibits global receptive fields, while the other methods display only local receptive fields. Although HiCARN theoretically allows for global coverage with its deep convolutional layers, this comes at the expense of a quadratic increase in computational cost. In contrast, all pixels are engaged in HiCMamba to highlight cross-pixel activation. The integration of the 2D selective scan mechanism and locally-enhanced feedforward neural network together ensure that the central pixel is primarily influenced by pixels along the cross, thereby facilitating both global and local dependency contexts.

Furthermore, to ensure a rigorous and fair comparison specifically between the attention and state space model mechanisms, we construct a baseline named “UNet-Transformer.” This baseline utilizes the exact same U-Net backbone as HiCMamba, with the only difference being the replacement of our holistic scan blocks (SS2D + LEFN) with standard Transformer blocks. By maintaining an identical macro-architecture, we eliminate interference from external architectural factors, allowing us to isolate and evaluate the intrinsic capability of Mamba versus Transformer in capturing long-range dependencies and managing computational scalability. As detailed in S3 Table of the supplementary materials, HiCMamba consistently outperforms the UNet-Transformer across all metrics on the K562 dataset, with a significant advantage in compartment score (0.6810 vs. 0.6046). This result empirically confirms that the state space model captures global genomic contexts (e.g., long-range chromosomal compartments) more effectively than standard self-attention mechanisms.

Moreover, experimental results reveal that a fundamental efficiency gap between the two architectures. In higher-resolution scenarios (e.g., 5kb or 1kb bin sizes), larger window sizes are required. As shown in S4 Table of the supplementary materials, we test input window sizes ranging from 40 to 320 bins on the same device. Due to the quadratic complexity (*O*(*N*^2^)) of self-attention, the UNet-Transformer suffers from drastic increases in peak memory usage and inference time, resulting in an ”Out Of Memory” error at the 320 window size. Conversely, HiCMamba exhibits linear scalability (*O*(*N*)), processing the 320-bin input with only approximately 1.2 GB of memory. Thus, HiCMamba offers a scalable solution that preserves long-range biological fidelity without the prohibitive computational cost associated with Transformer-based approaches.

In addition to comparison with Transformer-based architectures, we perform a comprehensive benchmark of parameters, MACs, peak GPU memory, and inference time against leading benchmark methods (i.e., HiCARN, HiCSR, HiCNN), as detailed in S4 Table of the supplementary materials. Notably, MACs are reported as a standard proxy for FLOPs to characterize computational complexity. To ensure a fair and rigorous comparison, all tests are conducted on a single GPU device (Tesla V100) across window sizes from 40 to 320. The inclusion of the 320×320 window size is critical, as it directly simulates high-resolution scenarios (e.g., 5kb or 1kb) where models must handle the increased computational load and long-range dependencies inherent in whole-genome interaction matrices. Beyond memory efficiency, Fig 3D demonstrates a critical advantage in algorithmic efficiency regarding MACs. The computational complexity of HiCMamba increases linearly as the input size grows. At the 320 resolution, HiCMamba requires only 38.8 giga multiply-accumulate operations (GMACs). In stark contrast, leading CNN-based methods impose a significantly heavier computational burden: HiCARN requires 140.6 GMACs, which is 3.6 times higher than our model, and HiCSR demands 241.3G MACs, representing a 6.2-fold increase. Most notably, HiCNN exhibits explosive computational cost at 1166.6 GMACs, representing a 30-fold increase over HiCMamba. These demonstrate that HiCMamba achieves superior feature extraction with a fraction of the computational budget required by dense convolution operations. Regarding inference time, we acknowledge that HiCMamba currently exhibits slightly higher latency than mature CNN baselines (e.g., HiCARN) at smaller input sizes. However, viewed alongside the MACs data, this is clearly an implementation-level rather than an algorithm-level limitation. CNNs benefit from over a decade of low-level operator optimization via libraries like cuDNN, whereas Mamba is a recent innovation with nascent kernel support [15]. Crucially, our significantly lower MACs count indicates that HiCMamba has a much higher theoretical speed ceiling. As hardware-aware optimizations for SSMs mature, HiCMamba is positioned to surpass CNNs in wall-clock speed, given that it performs significantly fewer underlying operations.

### HiCMamba shows generalization capability across different cell lines

To assess the real-world generalization capability of HiCMamba, we conduct cross-dataset validation using GM12878 and K562 cell lines. Specifically, the compared models are trained on one cell line and tested on the other, with results represented in Table 2. As expected, all methods showcase a slight performance decrease compared to the within-dataset evaluation (Table 1). However, HiCMamba consistently outperforms other models on the GM12878 cell line and maintains competitiveness on the K562 cell line.

**Table 2:** Cross-cell line performance evaluation of Hi-C resolution enhancement methods using GM12878 and K562 as independent test sets alternatively. Results highlighted in bold and underlined represent the best and second-best, respectively.

Methods	Trained on K562 and tested on GM12878		Trained on GM12878 and tested on K562	
	PCC	SRCC	PCC	SRCC
HiCSR [13]	0.5273	0.4644	0.4349	0.3693
HiCNN [11]	0.5832	0.5229	0.4883	0.3958
HiCARN [14]	0.5847	0.5232	0.4911	0.3929
HiCMamba (Ours)	${\text{0.6041}}_{+3.3\%}$	${\text{0.5449}}_{+4.2\%}$	${\text{0.5138}}_{+4.6\%}$	${\text{0.4123}}_{+4.2\%}$

We further introduce the IMR90 cell line as an independent test set to rigorously assess generalization on unseen data. We conduct evaluations where models trained on GM12878 or K562 are directly applied to IMR90, with results summarized in Table 3. Quantitative results highlight the robust generalization capability of HiCMamba, as it consistently outperforms state-of-the-art baselines in both scenarios. For instance, when trained on K562 and applied to IMR90, HiCMamba attains a PCC of 0.5474, significantly surpassing the 0.5284 achieved by the runner-up, HiCARN. This consistent performance indicates that HiCMamba learns universal 3D structural features rather than overfitting to specific cell-line patterns, demonstrating its robust generalization capability for real-world applications.

**Table 3:** Performance evaluation on IMR90 cell line using models trained on GM12878 and K562 respectively. Results highlighted in bold and underlined represent the best and second-best, respectively.

Methods	Trained on GM12878 and tested on IMR90		Trained on K562 and tested on IMR90	
	PCC	SRCC	PCC	SRCC
HiCSR [13]	0.4706	0.4074	0.4607	0.3700
HiCNN [11]	0.5262	0.4319	0.5261	0.4292
HiCARN [14]	0.5247	0.4272	0.5284	0.4277
HiCMamba (Ours)	${\text{0.5452}}_{+3.6\%}$	${\text{0.4445}}_{+2.9\%}$	${\text{0.5474}}_{+3.6\%}$	${\text{0.4445}}_{+3.6\%}$

### Ablation study validates the synergy of SS2D and LEFN

HiCMamba integrates the SS2D module and the LEFN module to simultaneously capture long-range dependencies and local patterns, providing a holistic view of chromatin interactions. To quantify the contribution of each model component, we conduct a systematic ablation study on the K562 dataset, comparing the full HiCMamba model against two variants: (1) ‘LEFN-only’ variant: retains the U-Net backbone and LEFN module but removes the SS2D module. (2) ‘SS2D-only’ variant: retains the U-Net backbone and SS2D module but removes the LEFN module. As shown in Table 4, the full HiCMamba model consistently achieves the highest performance, significantly outperforming both single-module variants. This superior performance stems from the distinct and complementary roles of the two core components. First, the SS2D module is critical for maintaining global chromosomal organization. In the ‘LEFN-only’ variant, the compartment score drops significantly from 0.6810 to 0.3082, exposing the inability of pure CNNs to capture long-range dependencies. Second, the LEFN module is essential for preserving local structural fidelity. In the ‘SS2D-only’ variant, the model suffers a substantial decline in the HiC-Spector score (from 0.9231 to 0.7471), underscoring the necessity of local convolutions for fine-grained pattern recovery. Collectively, these results validate the synergy between the global receptive field of SS2D and the local feature extraction of LEFN.

**Table 4:** Ablation results of each component of HiCMamba.

Method	PCC	SRCC	GenomeDISCO	HiC-Spector	Compartment
LEFN only	0.5090	0.4098	0.8328	0.6915	0.3082
SS2D only	0.5155	0.4127	0.8433	0.7471	0.6082
HiCMamba	**0.5219**	**0.4236**	**0.9310**	**0.9231**	**0.6810**

### HiCMamba enhances chromatin loop detection

**Fig 4:**
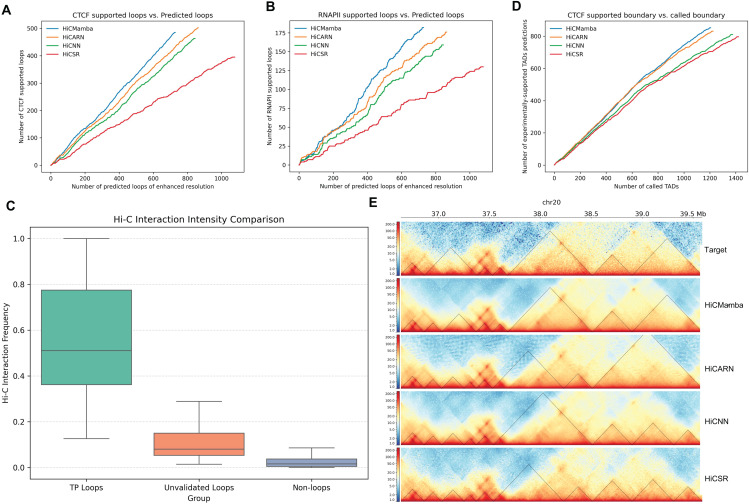
Comparison of chromatin loop and TAD annotations derived from enhanced contact maps by HiCMamba and compared methods. **(A-B)** Comparison of the number of predicted loops on enhanced contact maps against the number of CTCF-supported (A) and RNAPII-supported (B) loops identified through ChIA-PET. **(C)** Comparison of interaction frequencies among biologically validated ground-truth loops (supported by CTCF or RNAPII), unvalidated predictions (predicted by HiCMamba but not in CTCF/RNAPII lists), and a negative control set of randomly selected non-loop interactions matched for sample size and genomic distance. **(D)** Comparison of the number of predicted TAD boundaries on enhanced contact maps against the number of CTCF-supported TAD boundaries. **(E)** Example of TAD boundaries identified from the original high-coverage HiC map (top panel), and enhanced contact maps generated by four deep learning methods (botton four panels) within a region of Chromosome 20: 37.5-39.5Mb from the GM12878 dataset.

Accurate chromatin loop identification is crucial for understanding gene regulation and disease mechanisms [32,33]. This section investigates whether HiCMamba enhances chromatin loop detection. We employ the off-the-shelf loop annotation tool HiCCUPS [9] to contact maps generated by HiCMamba and other deep learning methods using the GM12878 dataset. We quantify the accuracy of loop predictions using the ‘proportion’ metric, defined as the ratio of experimentally validated loops to the total number of computationally predicted loops, following Zhang et al. [34]. We compared the predicted loops against the ground-truth set of CTCF and RNAPII-supported loops derived from the Chromatin Interaction Analysis by Paired-End Tag Sequencing (ChIA-PET) [35] data (obtained from GEO under accession number GSE72816 [17]). Table 5, Fig 4A and 4B showcase the number of predicted loops and their overlap with ChIA-PET-validated loops. HiCMamba outperforms the runner-up with a precision improvement of 8.4% for CTCF-supported loops and 4.6% for RNAPII-supported loops. These results suggest that HiCMamba generates more fine-grained contact maps, which are crucial for accurate chromatin loop identification.

**Table 5:** Comparison of CTCF and RNAPII-supported ChIA-PET loops with loops detected in enhanced contact maps generated by various deep learning methods.

	Methods	Matched	Predicted	Proportion
CTCF	HiCMamba (Ours)	485	730	66.4%
	HiCARN [14]	502	865	58.0%
	HiCNN [11]	463	846	54.7%
	HiCSR [13]	395	1079	36.6%
RNAPII	HiCMamba (Ours)	182	730	24.9%
	HiCARN [14]	176	865	20.3%
	HiCNN [11]	159	846	18.8%
	HiCSR [13]	130	1079	12.0%

We perform comprehensive experiments to investigate the biological nature of the unvalidated loops identified by HiCMamba. We cross-validate our predicted loops against both CTCF and RNAPII. Specifically, a complementary relationship is revealed that loops unverified by CTCF may be validated by other regulators like RNAPII (1.78%), and conversely, loops lacking RNAPII support are frequently validated by CTCF alone (43.29%). Consequently, 68.22% of the predictions are supported by at least one of these regulators, suggesting that interactions missed by a single marker often represent biologically relevant structures mediated by alternative mechanisms. Crucially, to ascertain whether the remaining unvalidated loops represent aberrant structures, we interrogate their physical interaction intensity within the high-resolution ground truth Hi-C maps. We conduct a comparative analysis involving validated ground-truth loops, unvalidated predictions (predicted by HiCMamba but not in CTCF/RNAPII lists), and a negative control set of randomly selected interactions strictly matched for sample size and genomic distance. As illustrated in Fig 4C, the unvalidated loops exhibit significantly higher interaction frequencies compared to the non-loops group. These results indicate that HiCMamba preserves genuine physical patterns while minimizing the introduction of artifacts, suggesting that these unvalidated structures likely represent bona fide loops mediated by other regulators.

### HiCMamba is more accurate in TAD boundary annotation

Topologically associating domains (TADs) are chromosomal regions formed by chromatin loop extrusion, with boundaries demarcated by architectural proteins [36]. These structures are essential for pinpointing functionally relevant sub-regions such as subTADs and microTADs. To assess the utility of HiCMamba in TAD annotation, we use HiCExplorer [37] to reconstruct the TADs boundaries on enhanced contact maps generated by HiCMamba and benchmarking tools on GM12878. CTCF ChIP-seq peaks are utilized to measure the precision of the recovered TADs [34]. Table 6 and Fig 4D compare the number of predicted TADs and the number of CTCF ChIP-seq supported boundaries across different methods. HiCMamba demonstrates superior performance with the highest precision values among all evaluated methods, and surpasses HiCARN, HiCNN, and HiCSR by 2.9%, 11.7%, and 14.6%, respectively.

**Table 6:** Comparison of CTCF ChIP-seq supported boundaries with those detected in enhanced contact maps generated by deep learning methods.

Methods	Matched	Predicted	Proportion
HiCMamba (Ours)	852	1207	70.6%
HiCARN [14]	831	1226	67.7%
HiCNN [11]	810	1376	58.9%
HiCSR [13]	796	1422	56.0%

Fig 4E provides a visual comparison of predicted TAD boundaries and CTCF ChIP-seq supported boundaries within the region of chromosome 20: 36.5Mb-39.5Mb. Although other methods demonstrate partial TAD reconstruction, HiCMamba consistently identifies more accurate TAD boundaries. This enhanced accuracy highlights the effectiveness of HiCMamba in recovering high-coverage Hi-C contact maps, enabling a more precise and comprehensive analysis of TAD structures.

Moreover, we assess the impact of downsampling ratios (1/32, 1/64, and 1/100) on biological structures (TADs and loops), as shown in S1B and S1C Fig of the supplementary materials. We observe that TADs are remarkably resilient to sparsity: as the downsampling ratio becomes more extreme (from 1/16–1/100), the number of recovered TADs decreases only slightly, from 852 to 719. This suggests that HiCMamba can reliably reconstruct large-scale chromatin domains even from highly sparse data. In contrast, the fine-scale features, chromatin loops, are more sensitive. The recovery of experimentally validated loops drops from 485 (at 1/16) to 166 (at 1/100), indicating that while global structures are preserved, fine-grained point-to-point interactions become increasingly difficult to distinguish from noise at extreme sparsity levels. Overall, HiCMamba maintains consistent and robust performance across the varying downsampling levels.

### Correlation between the 3D genome and epigenomics

The organization of higher-order chromatin is essential for gene regulation and cellular homeostasis [6,38]. We investigate the relationship between 3D genome structure and epigenomic features (i.e., SEs, SE elements, ChIP-seq signals, and methylation signals), which are integral to understanding gene expression and chromatin organization.

**Fig 5:**
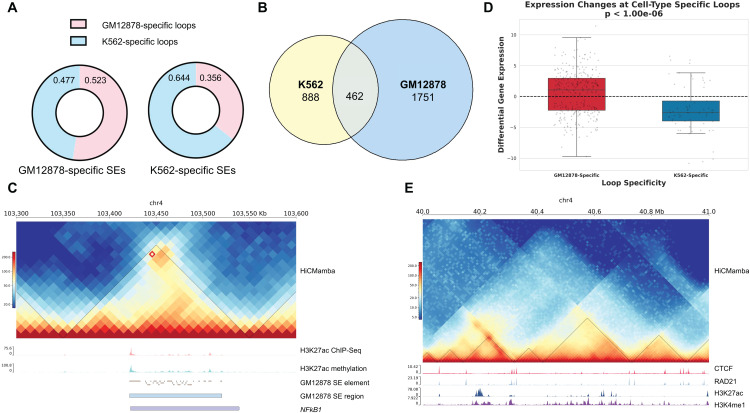
Epigenomic features and 3D genome structures are essential for gene expression. **(A)** Structure weighted scores of cell type-specific loops versus cell type-specific super-enhancers (SEs). **(B)** Venn Diagram of Differentially Expressed Genes in GM12878 and K562 Cell Lines. **(C)** 3D genome structure and epigenomic features around the *NFKB1* gene locus. **(D)** Global concordance between cell-type-specific loops and differential gene expression. The x-axis categorizes genes by their association with cell-type-specific loops, while the y-axis represents the *log*_2_ fold change of gene expression (GM12878 vs. K562). **(E)** A snapshot of TAD and ChIP-seq signals of transcription factors on chromosome 4: 40Mb-41Mb.

First, we conduct a systematic comparison of cell-type-specific loops and TADs across all methods to evaluate their ability to capture cell identity. To quantify this, we employ the cell-type-specific structure weighted scoring metric, associating loops with cell-type-specific SEs, as well as TADs with CTCF binding sites. As detailed in S1 and S2 Table of the supplementary materials, HiCMamba demonstrates highly competitive performance in capturing cell identity. Particularly on the K562 cell line, which exhibits greater data sparsity, HiCMamba achieves a specificity score of 0.644 on the K562 cell line, significantly outperforming the second-best method, HiCNN (0.560). Regarding TAD boundary specificity, HiCMamba leads on the GM12878 dataset with a score of 0.629, surpassing HiCSR (0.589). These results indicate that HiCMamba is highly robust in preserving cell-type-specific features that define cellular identity. Fig 5A showcases a representative pattern where cell-type-specific SEs exhibit a strong association with the identified loops. These findings emphasize the critical role of cell-type-specific loops in their respective functions in concordance with previous research [39].

Further analysis of differentially expressed genes (DEGs) between GM12878- and K562-specific loops (Fig 5B) reveals functional links to 3D genome structure. For example, the acetylation cycle of cohesin, which modulates chromatin loop length through a *PDS5A*-mediated brake mechanism [40], is one such process involving overlapping DEGs. Similarly, the gene *DNAH3*, located adjacent to a specific deletion, exhibits chromatin interactions with enhancer elements within the deleted region [41]. These examples highlight the connection between DEGs and 3D genome organization. Moreover, cell-type-specific DEGs tend to be involved in cell differentiation. For instance, *NFKB1* shows significantly higher expression in GM12878 compared to K562, with associated loops being exclusively found in GM12878. This observation aligns with the constitutive activation of *NFKB1* pathways in the GM12878 lymphoblastoid B-cell line [42]. Fig 5C depicts the loop architecture associated with *NFKB1* expression, revealing concomitant increases in H3K27ac ChIP-seq and methylation peaks within the loop region. Additionally, GM12878-specific SE and SE elements are observed around these regions, suggesting a potential regulatory role in this context. Notably, the distribution of SE elements aligns with the ChIP-seq and methylation peaks of H3k27ac. These findings are corroborated by Zhao et al. [43], who demonstrated the essential role of *NFKB1* subunits, enriched at active enhancers marked by H3K27ac signals, in B cell development and function. We then quantify how the enhanced 3D structural changes specifically explain the differences in gene expression at the *NFKB1* locus, as shown in S2 Fig of the supplementary materials. The loop exhibits a robust interaction score in GM12878, coinciding with high gene expression. In contrast, the interaction score in K562 shows a more than 4-fold decrease, which closely parallels a dramatic nearly 20-fold downregulation in *NFKB1* expression. This analysis is further extended to a global scale to demonstrate that this finding represents a general regulatory principle. Specifically, we categorize genes based on their association with cell-type-specific loops and quantify their differential expression as the log2 fold change between GM12878 and K562. As illustrated in Fig 5D, the results reveal a striking functional divergence: genes anchored to GM12878-specific loops exhibit predominantly positive log-fold changes (signifying upregulation in GM12878), whereas those linked to K562-specific loops display negative values (signifying upregulation in K562). This robust global concordance confirms that the 3D structural enhancements provided by HiCMamba effectively capture the functional drivers of cell-type-specific gene regulation.

Finally, Fig 5E illustrates the distinct enrichment patterns of various epigenomic features within and around TADs. CTCF and RAD21 ChIP-seq signals are enriched at the TAD boundaries, whereas the H3K27ac and H3K4me1 signals are enriched within the TADs, consistent with previous studies [44–46]. Altogether, HiCMamba can effectively recover chromatin interaction patterns such as loops and TADs. These 3D genome structures are intricately associated with various epigenomic features, contributing to the transcriptional regulation of cell-type-specific genes.

## Discussion

Three-dimensional chromatin structures, e.g., topologically associating domains (TADs) and loops, derived from Hi-C data are essential for deciphering the intricate relationship between chromatin organization and transcription regulation. Obtaining high-resolution Hi-C data poses significant technical and financial challenges, leading to the prevalence of low-resolution contact maps that hinder accurate interaction frequency estimations. In this work, we presented a novel framework HiCMamba based on state space modeling, for the efficient and accurate in-silico enhancement of Hi-C contact maps. To the best of our knowledge, HiCMamba is the first model to harness a state space model for enhancing Hi-C resolution.

Specifically, HiCMamba combined the strengths of UNet architecture and a novel holistic scan block to enable effective multi-scale contact map processing. The holistic scan block comprised an SS2D module, which leverages Mamba’s long-range modeling capabilities for comprehensive feature extraction, and an LEFN module, which optimizes information flow for enhanced accuracy and efficiency. Evaluations on GM12878 and K562 Hi-C datasets demonstrated the superior performance of HiCMamba compared to the state-of-the-art deep learning methods. It achieved high-quality recovery results at a remarkably low computational cost, requiring only 25% of the resources compared to the second-best method. Cross-cell line experiments further validated its robust generalization capabilities. Moreover, unlike other methods confined to local receptive fields, HiCMamba features global receptive fields, enabling the efficient representation of distant genomic loci. Importantly, HiCMamba-enhanced contact maps yielded 3D genome structures, TADs and loops, with significantly fewer false positives compared to other methods. Our analysis also revealed a strong association between cell-type-specific SEs and loops, highlighting the importance of these structures in cell-type-specific functions. Furthermore, we observed an intricate interplay between 3D genome organization and various epigenomic features, suggesting a combined role in regulating cell-type-specific gene expression.

Although HiCMamba demonstrated its effectiveness in Hi-C contact maps enhancement, several avenues for future improvement exist. First, HiCMamba processes the contact maps as images, akin to an image restoration task. However, incorporating DNA sequence data, which has shown promise in predicting 3D genome structures [47, 48], could provide a more comprehensive understanding of locus-specific contact patterns. Additionally, while extending HiCMamba’s capacity (now at 10kb resolution) to analyze higher resolutions is feasible, it would demand more fine-grained Hi-C data and increased computational resources. This work makes a significant contribution by demonstrating the potential of state space models for resolution enhancement of Hi-C contact maps, paving the way for future advancements in the field.

## Supporting information

S1 Fig Quantitative evaluation of HiCMamba performance across varying data sparsity levels. (**A**) PCC between enhanced and ground-truth maps across varying genomic distances for GM12878 at different downsampling ratio. (**B**) Resilience of Topologically Associating Domain (TAD) recovery across downsampling ratios. (**C**) Sensitivity of chromatin loop recovery to data sparsity.
(DOCX)

S2 Fig Quantitative correlation between chromatin loop intensity and gene expression at the *NFKB1* locus. (DOCX)

S1 Table Quantitative evaluation of cell-type specific loops recovered by various methods. (DOCX)

S2 Table Quantitative evaluation of cell-type specific TADs recovered by various methods. (DOCX)

S3 Table Comparison results between HiCMamba and UNet-Transformer. (DOCX)

S4 Table Parameters, floating-point operations (FLOPs), peak GPU memory usage, and inference time across different methods and different input size. OOM represents Out-Of-Memory.
(DOCX)
