# Supplementary material for: HiCMamba: Enhancing Hi-C resolution and identifying 3D genome structures with state space modeling
Source: PLoS Comput Biol. 2026 Mar 24;22(3):e1014057. doi: 10.1371/journal.pcbi.1014057 (PMC13012732; doi:10.1371/journal.pcbi.1014057)

**S1 Fig.** Quantitative evaluation of HiCMamba performance across varying data sparsity levels. (**A**) PCC between enhanced and ground-truth maps across varying genomic distances for GM12878 at different downsampling ratio. (**B**) Resilience of Topologically Associating Domain (TAD) recovery across downsampling ratios. (**C**) Sensitivity of chromatin loop recovery to data sparsity.


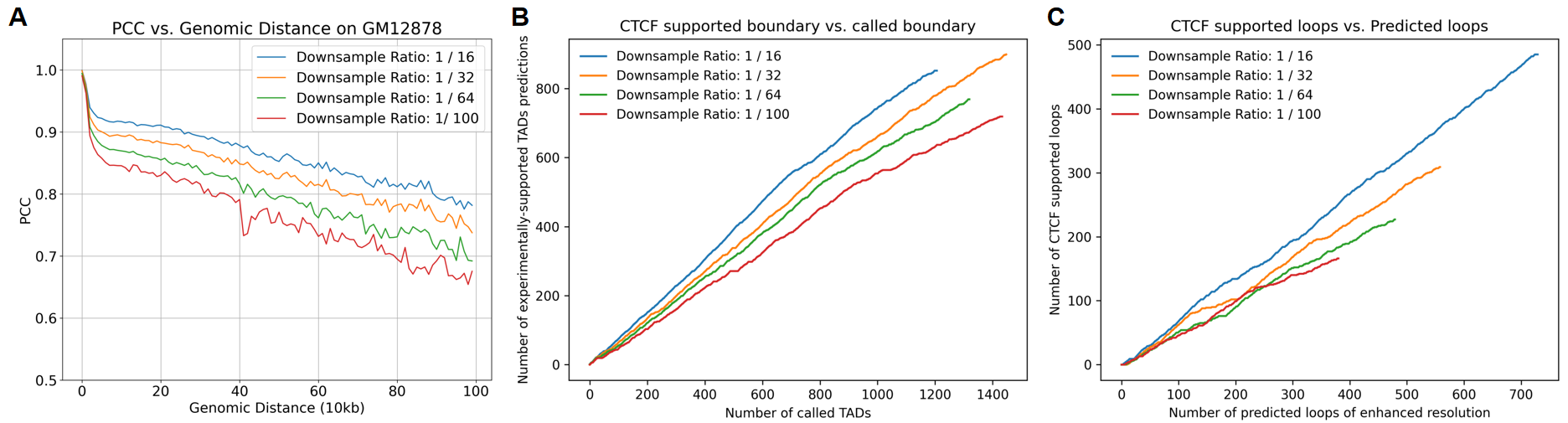

Supplement: S1 Fig — (A) PCC between enhanced and ground-truth maps across varying genomic distances for GM12878 at different downsampling ratio. (B) Resilience of Topologically Associating Domain (TAD) recovery across downsampling ratios. (C) Sensitivity of chromatin loop recovery to data sparsity. (DOCX) [file pcbi.1014057.s001.docx]
