# Supplementary material for: HiCMamba: Enhancing Hi-C resolution and identifying 3D genome structures with state space modeling
Source: PLoS Comput Biol. 2026 Mar 24;22(3):e1014057. doi: 10.1371/journal.pcbi.1014057 (PMC13012732; doi:10.1371/journal.pcbi.1014057)

**S2 Fig.** Quantitative correlation between chromatin loop intensity and gene expression at the *NFKB1* locus.


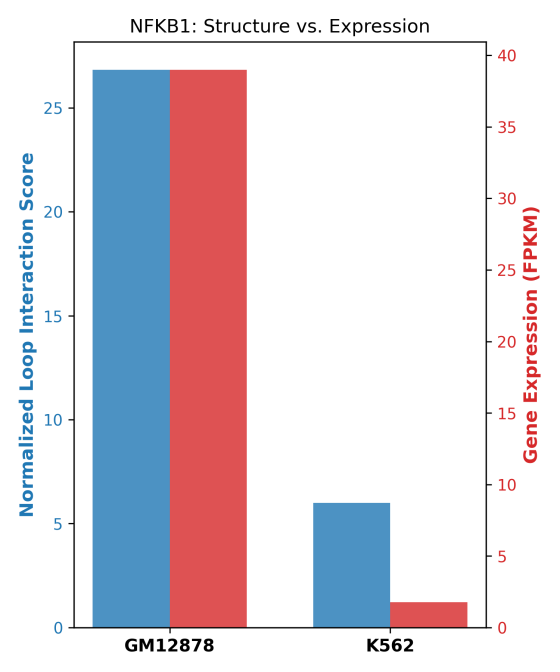

Supplement: S2 Fig — (DOCX) [file pcbi.1014057.s002.docx]
