# Supplementary material for: HiCMamba: Enhancing Hi-C resolution and identifying 3D genome structures with state space modeling
Source: PLoS Comput Biol. 2026 Mar 24;22(3):e1014057. doi: 10.1371/journal.pcbi.1014057 (PMC13012732; doi:10.1371/journal.pcbi.1014057)
Supplement: S1 Table — (DOCX) [file pcbi.1014057.s003.docx]

**S1 Table**. Quantitative evaluation of cell-type specific loops recovered by various methods.

| Method | $W_{GM12878}^{GM12878}$ | $W_{K562}^{K562}$ |
| --- | --- | --- |
| HiCSR | 0.531 | 0.493 |
| HiCNN | 0.513 | 0.560 |
| HiCARN | **0.546** | 0.537 |
| HiCMamba | 0.523 | **0.644** |
