# Supplementary material for: HiCMamba: Enhancing Hi-C resolution and identifying 3D genome structures with state space modeling
Source: PLoS Comput Biol. 2026 Mar 24;22(3):e1014057. doi: 10.1371/journal.pcbi.1014057 (PMC13012732; doi:10.1371/journal.pcbi.1014057)
Supplement: S2 Table — (DOCX) [file pcbi.1014057.s004.docx]

**S2 Table**. Quantitative evaluation of cell-type specific TADs recovered by various methods.

| Method | $W_{GM12878}^{GM12878}$ | $W_{K562}^{K562}$ |
| --- | --- | --- |
| HiCSR | 0.589 | **0.580** |
| HiCNN | 0.543 | 0.541 |
| HiCARN | 0.572 | 0.548 |
| HiCMamba | **0.629** | 0.561 |
