# Supplementary material for: HiCMamba: Enhancing Hi-C resolution and identifying 3D genome structures with state space modeling
Source: PLoS Comput Biol. 2026 Mar 24;22(3):e1014057. doi: 10.1371/journal.pcbi.1014057 (PMC13012732; doi:10.1371/journal.pcbi.1014057)
Supplement: S3 Table — (DOCX) [file pcbi.1014057.s005.docx]

**S3 Table**. Comparison results between HiCMamba and UNet-Transformer.

| Method | PCC | SRCC | GenomeDISCO | HiC-Spector | Compartment |
| --- | --- | --- | --- | --- | --- |
| UNet-Transformer | 0.5136 | 0.4055 | 0.9116 | 0.9152 | 0.6046 |
| HiCMamba | **0.5219** | **0.4236** | **0.9310** | **0.9231** | **0.6810** |
