# Supplementary material for: HiCMamba: Enhancing Hi-C resolution and identifying 3D genome structures with state space modeling
Source: PLoS Comput Biol. 2026 Mar 24;22(3):e1014057. doi: 10.1371/journal.pcbi.1014057 (PMC13012732; doi:10.1371/journal.pcbi.1014057)
Supplement: S4 Table — OOM represents Out-Of-Memory. (DOCX) [file pcbi.1014057.s006.docx]

**S4 Table**. Parameters, floating-point operations (FLOPs), peak GPU memory usage, and inference time across different methods and different input size. OOM represents Out-Of-Memory.

| Model | Params (M) | Input Size | MACs(G) | Peak Memory Usage (MB) | Inference Time (ms) |
| --- | --- | --- | --- | --- | --- |
| HiCMamba | 1.78 | 40 | 0.6 | 26.1 | 19.1 |
|  |  | 80 | 2.4 | 83.8 | 19.7 |
|  |  | 160 | 9.69 | 318.7 | 21.1 |
|  |  | 320 | 38.7 | 1240.5 | 86.4 |
| UNet-Transformer | 1.28 | 40 | 0.5 | 48.4 | 8.9 |
|  |  | 80 | 2.1 | 644.0 | 15.0 |
|  |  | 160 | 8.1 | 10061.2 | 226.9 |
|  |  | 320 | OOM | OOM | OOM |
| HiCSR | 2.26 | 40 | 4.98 | 32.0 | 5.3 |
|  |  | 80 | 17.1 | 48.4 | 7.9 |
|  |  | 160 | 62.9 | 106.4 | 9.3 |
|  |  | 320 | 241.3 | 337.1 | 33.3 |
| HiCNN | 4.17 | 40 | 18.2 | 100.7 | 8.0 |
|  |  | 80 | 72.9 | 213.5 | 9.3 |
|  |  | 160 | 291.7 | 770.1 | 34.2 |
|  |  | 320 | 1166.6 | 2995.8 | 146.1 |
| HiCARN | 1.38 | 40 | 2.2 | 44.5 | 8.5 |
|  |  | 80 | 8.7 | 88.9 | 9.8 |
|  |  | 160 | 35.1 | 27.8 | 12.9 |
|  |  | 320 | 140.6 | 991.9 | 26.4 |
